# Intracellular accumulation of Praziquantel in T lymphoblastoid cell lines, CEM (parental) and CEMvbl(P-gp-overexpressing)

**DOI:** 10.1186/s40360-016-0079-4

**Published:** 2016-08-14

**Authors:** Gabriel Kigen, Geoffrey Edwards

**Affiliations:** 1 Department of Pharmacology and Toxicology, Moi University School of Medicine, P.O. Box 4606, 30100 Eldoret, Kenya; 2 Department Molecular and Clinical Pharmacology, University of Liverpool, Liverpool, L69 3GE UK

**Keywords:** Praziquantel, P-glycoprotein, Characterization, Pharmacokinetics

## Abstract

**Background:**

Praziquantel (PZQ) is an antihelminthic drug whose P-glycoprotein (P-gp) substrate specificity has not been conclusively characterized. We investigated its specificity by comparing its *in vitro* intracellular accumulation in CEM (parental), and CEMvbl cells which over express P-gp, a drug efflux transporter. Saquinavir (SQV), a known substrate of efflux transporters was used as control.

**Methods:**

A reversed phase liquid chromatography method was developed to simultaneously quantify PZQ and SQV in cell culture media involving involved a liquid - liquid extraction followed by ultra-high performance liquid chromatography using a Hypurity C_18_ column and ultraviolet detection set at a wavelength of 215 nm. The mobile phase consisted of ammonium formate, acetonitrile and methanol (57:38:5 v/v). Separation was facilitated via isocratic elution at a flow rate of 1.5 ml/min, with clozapine (CLZ) as internal standard. This was validated over the concentration range of 1.6 to 25.6 μM for all analytes. Intracellular accumulation of SQV in CEMvbl was significantly lower compared to that in CEM cells (0.1 ± 0.031 versus 0.52 ± 0.046, *p* = 0.03 [*p* <0.05]).

**Results:**

Accumulation of PZQ in both cell lines cells were similar (0.05 ± 0.005 versus 0.04 ± 0.009, *p* = 0.4) suggesting that it is not a substrate of P-gp in CEM cells. In presence tariquidar, a known inhibitor of P-gp, the intracellular accumulation of SQV in CEMvbl cells increased (0.52 ± 0.068 versus 0.61 ± 0.102, *p* = 0.34 in CEM cells and 0.09 ± 0.015 versus 0.56 ± 0.089, *p* = 0.029 [*p* < 0.05] in CEMvbl cells). PZQ did not significantly affect the accumulation of SQV in either CEM (0.52 ± 0.068 versus 0.54 ± 0.061, *p* = 0.77), or in CEMvbl cells (0.09 ± 0.015 versus 0.1 ± 0.031, *p =* 0.89) cells compared to tariquidar, implying that PZQ is not an inhibitor of P-gp in CEMvbl cells.

**Conclusions:**

PZQ is neither a substrate nor an inhibitor of the efflux drug transporter P-gp in T-lymphoblastoid cells, CEM and CEMvbl. We also report a simple, accurate and precise method for simultaneous quantification of PZQ and SQV.

## Background

Praziquantel (PZQ) is a broad spectrum antihelminthic drug used in the mass treatment of lymphatic filariasis and schistosomiasis which afflicts over 243 million people [1, 2]. It is widely used because of its low cost and efficacy. It is also used in combination with albendazole for the treatment of neurocystercosis [3]. The three conditions constitute serious public health problems in the developing countries of Africa, Asia and Latin America [4, 5]. The same regions bear the burden of HIV/AIDS, especially sub-Saharan Africa. Praziquantel is therefore quite often co-administered with several other drugs including antiretroviral drugs [ARVs] [6–9]. The co-administration may give rise to drug-drug interactions that could influence the treatment outcomes, which in some instances may require dosage adjustments in order to prevent toxicity or resistance [10–12]. Despite its widespread use, there is currently limited information regarding PZQ’s mechanisms of action at molecular level, and a lot more research is required in its pharmacokinetics in order to prevent resistance [13].

A possible mechanism for interactions between drugs results from the modulation of the efflux drug transporter, P-gp. P-gp functions to transport drugs from the intracellular to the extracellular domain, often against concentration gradients. The inhibition or potentiation of the transporter function will therefore have an impact on the cellular accumulations of the drug’s efficacy [14, 15]. Several drugs are substrates and/or inhibitors of efflux drug transporters and metabolic enzymes (especially CYP 3A4). Among the ARVs, protease inhibitors (PIs) including SQV are known to be substrates of P-gp, ABCC 1 and ABCC 2 [14, 16, 17]. With regards to drug transporter specificity, PZQ has not been conclusively characterized to date. In one study, the authors concluded that PZQ is an inhibitor of P-gp without being a substrate [18], whereas those in a related study indicated that PZQ did not show potential for interacting with cellular efflux pumps despite being a highly permeable substance [19, 20]. PZQ has also been reported to inhibit the transport by SMDR2, a P-gp orthologue from *S. mansoni* [21], and P-gp has also been postulated to play a role in the resistance of PZQ [22–24].

The intracellular accumulation of drugs is controlled by several factors including ion trapping, lipophilicity and plasma protein binding, as well as influx and efflux transporters. With regards to PIs, drug transporters P-gp, ABCC1, ABCC2 and BCRP play an important role in their accumulation [25, 26]. Being an efflux transporter, P-gp transports PIs from the intracellular to extracellular compartments, and the differences in their accumulation may be used to study their pharmacokinetics [15]. CEM cells treated with vinblastine (CEMvbl) overexpress P-gp [27], and the comparison of the *in vitro* accumulation of PIs in CEM parental and CEMvbl cells has been used to investigate the effects of active transport [15, 28]. Previous studies in our laboratory have investigated the intracellular accumulation of SQV in T-lymphoblastoid cells, CEM parental and CEMvbl cells [25].

Our study had a double objective; to develop a suitable assay method for simultaneous quantification of both PZQ and SQV and to characterize PZQ with regards to substrate specificity of the transporter P-gp. In order to ascertain whether PZQ is a substrate of P-gp, its intracellular accumulation in CEM parental and CEMvbl cells which overexpress P-gp were compared to that of SQV, a known substrate of P-gp [15, 25, 28]. To determine whether it is an inhibitor, the accumulation of SQV in CEMvbl cells was compared to its accumulation in presence of PZQ, and in presence of a known inhibitor, tariquidar (XR). A reversed phase liquid chromatography method was validated for simultaneous quantification of both PZQ and SQV in cell culture media and cell pellets.

## Methods

### Chemicals and reagents

SQV (Formula weight, 670.86) was donated by Roche Pharmaceuticals (Welwyn Garden City, UK). PZQ (cat. no. P4668, formula weight, 312.41); Clozapine [CLZ] (cat. no. C6305, Formula Weight, 326.82); Dulbecco’s Modified Eagle’s Medium, [DMEM] (cat. no. D6249, containing 4500 mg/L glucose, 4 mM L-glutamine and 110 mg/L sodium pyruvate); Hanks Balanced Salt Solution [HBSS] (cat. no. H8264, modified with sodium bicarbonate, without phenol red, liquid, sterile-filtered, cell culture tested); Roswell Park Memorial Institute medium [RPMI] (cat. no. R8758)]; Foetal Bovine Medium, FBS (cat. F7524) and Trypsin-EDTA solution were purchased from Sigma Chemical Co. (Poole, UK). Acetonitrile (ACN) and methanol (MeOH) were purchased from VWR international (Leicestershire, UK); whereas diethyl ether was purchased from Fisher Scientific, (Leicestershire, UK). Tariquidar was kindly donated by Xenova Group plc (Berkshire, UK). All the other chemicals used were of analytical or HPLC grade. Deionised water used to prepare the solutions or mobile phase was purified in an Elga DV 25 pure lab option system (Elga, High Wycombe, Bucks, and UK). T-lymphoblastoid cell lines, CEM and CEMvbl cells were gifts from Dr. R. Davey (University of Queensland, Australia), and the cells were counted using a Nucleo Counter (ChemoMetec, Denmark) cell counter.

### Equipment and chromatographic conditions

The high performance liquid chromatography (HPLC) consisted of a Dionex (Dionex Softron GmbH, Germany) HPLC system with a P 680 pump, an ASI-100 automated sample injector and a UVD 1704 detector. A 250 μl injector with a 20 μl loop was used. Reversed-phase-liquid chromatography was carried out using a Hypurity™ C_18_ analytical column, 5 μm × 4.6 mm (Thermo Electron Corporation, Runcorn, UK 22105-154630). A column guard (Thermo electron 60140-412) was used to protect the analytical column. The ultraviolet detector was set to monitor at 215 nm wavelength. The mobile phase for the analysis was composed of ammonium formate 20 mM (pH = 4.2), ACN and MeOH (57:38:5 v/v), and was prepared fresh for each assay. The separation was facilitated via isocratic elution at 1.5 ml/min flow rate and the run time was eight minutes for each separation. 20 μl of the samples was injected for each run by means of an automated injector. The peak area ratios for the calibration curves and quantification were obtained and analyzed using Chromelon software (version 6.5).

### Preparation of stock solutions, calibrators, quality controls and internal standard

#### Stock solutions

Stock ammonium formate buffer was prepared by dissolving ammonium formate in deionised water to obtain a final concentration of 1 M (63.06 mg/ml). The pH was then adjusted by the addition of formic acid to 4.2 M, and stored at room temperature. This was stable for use for up to 6 months. Stock standard solutions of SQV, PZQ and the internal standard (IS), clozapine were prepared by dissolving the various solutions of the analyte in MeOH to obtain a final concentration of 1 mg/ml. They were then capped, labeled and stored at 4 °C until use.

#### Working solutions

Working ammonium formate buffer (20 mM [1.26 mg/ml]) was prepared by diluting the stock buffer with deionised water, 1:50 (v/v). Working stock standard solutions (100 μl each) of PZQ and SQV were prepared by appropriate dilution in DMEM media over a concentration range of 1.6 to 25.6 μM in duplicate (six concentration levels). Quality control (QC) samples (100 μl) were prepared by diluting the stock solutions of the analytes to give QC concentrations of 6.4 (LQC), 12.8 (MQC) and 19.2 μM (HQC). Sufficient volume of the working internal standard (5 μg/ml) was prepared fresh for each assay in a serial dilution of the stock CLZ solution in MeOH, first as 1 in 10 to give 10 μg/ml followed by 1 in 2.

#### Calibration curves

Working stock standard solutions of PZQ and SQV were prepared by appropriate dilution in DMEM media. For the construction of the calibration curve, 100 μl of the calibration standards were prepared by serial dilution of the stock SQV/PZQ solutions in DMEM (in duplicate), after thawing the samples resulting in seven concentration levels of 0, 1.6, 3.2, 6.4, 12.8, 19.2 and 25.6 μM in 10 ml labelled glass tubes (Table 1). Quality control samples (100 μl) were prepared by thawing the stock QC samples and pipetting into separate 10 ml labelled glass tubes (in duplicate) to give concentrations of 6.4 (LQC), 12.8 (MQC) and 19.2 μM (HQC).

**Table 1:** Concentrations of the standards and QCs

Tube No.	Levels	SQV/PZQ Conc.(μM)	Stock(μl)	DMEM(μl)
1,2	1	0	0	100
3,4	2	1.6	6.25	93.75
5,6	3	3.2	12.5	87.5
7,8	4	6.4	25	75
9,10	5	12.8	50	50
11,12	6	19.2	75	25
13,14	7	25.6	100	0
	Total		268.75(μl)	431.25(μl)
	QCs			
15,16	LQC	6.4	100	0
17,18	MQC	12.8	100	0
19,20	HQC	19.2	100	0

### Extraction procedure

Before the analysis, 100 μl of the calibration standards and QCs were pipetted in duplicate into 10 ml labeled glass tubes. 20 μl of 5 μg/ml of internal standard CLZ and 2mls of the extraction solvent diethyl ether were then added to each individual sample using a Finn repeater pipette. The tubes were then capped and tumbled for 30 min using a rotary mixer and then centrifuged for 5 min at 4000 × *g*. The aqueous phase was then frozen in a cryogenic bath and the solvent phases transferred to correspondently labelled 5 ml clean tubes. This was followed by evaporation of the solvent to dryness using a centrifugal rotary evaporator (Jouan RC.10.10). The residues were reconstituted by the addition of 100 μl mobile phase and all the tubes vortexed. 100 μl of the samples were then aliquoted into autosampler vials, placed in corresponding numbered wells of the autosampler tray and securely capped. They were then centrifuged for 4 min at 4000 × *g*, and injected into the HPLC column (20 μl).

### Method validation

The validation of the method was carried out as per the FDA guidelines.

#### Linearity

In order to evaluate the linearity of the assay, ten six-point (1.6, 3.2, 6.4, 12.8, 19.2 and 25.6 μM) calibration curves were analyzed on separate days to determine the concentrations for each sample. The samples were extracted as described on section “Extraction procedure” and the standard curves were plotted as the peak area ratio (PAR) of the respective compound to the internal standard versus the concentration. The curves were obtained by the use of DMEM spiked with each of the six concentrations of both SQV and PZQ on the same run, and each point in the calibration curve run in duplicate. To assess linearity, the line of best fit was then determined by least squares regression.

#### Limit of quantification

The lowest limit of quantification (LLQ) for each drug was the minimal concentration that was within the range of the nominal concentration, with the acceptance criteria for each calculated standard concentration limited to not more than 20 % deviation from the nominal value. Calibration curves were established with standard solutions for the concentration points of 1.6, 3.2, 6.4, 12.8, 19.2 and 25.6 μM, all analyzed in quadruplicate.

#### Limit of detection

To determine the limit of detection (LLD), quadruplicate standard solutions for the concentration points of 1.6, 0.8, 0.4, 0.2, 0.1 and 0.05 μM in were analyzed, and the peak areas of the respective drug concentration compared to that in DMEM (blank). The lowest concentration that produced a signal three or more times above the noise level of a blank preparation was designated as the limit of detection (LOD).

#### Accuracy and precision

Inter-day accuracy and precision ware evaluated by comparing ten replicate low, medium and high QC levels that were analyzed on different days. The intra-day accuracy and precision was determined by the analysis of the three concentrations of the QC samples in six replicates within the same day, evaluated in duplicate. The obtained results were expressed as relative standard deviations to the mean and the Coefficient of variation (CV) expressed as a percentage; that is CV = (SD/Mean) × 100. The accuracy and precision of the analytical method was based on the fact that the relative standard deviation of each concentration should be within ± 20 % of the nominal concentration.

#### Recovery

The recovery or extraction efficiency of the analyte after the liquid-liquid extraction was determined by comparing the peak areas of six replicate samples of the QCs of each compound in extracted DMEM to those of non-processed standard solutions (standard solutions spiked in mobile phase). It was expressed as a percentage of the peak area of the extracted drug to that injected in MP.

#### Specificity, selectivity and stability

The separation from endogenous compounds was investigated by analyzing six different samples of DMEM. Short and long term stability was investigated by comparing the peak areas of six freshly prepared QC assay samples to those of the freeze-thawed samples, and it was expressed as a percentage.

### Drug accumulation experiments

#### Cell culture

The parental cell line was CEM (T-lymphoblastoid cell line) was used as the parental cell line. CEMvbl cells that have increased expression of P-gp were selected under vinblastine [25]. The selection of drug resistant cells was carried out by using increasing concentrations of vinblastine up to 100 ng/mL. CEM and CEMvbl Cells were cultured in 175 cm^2^ flasks containing DMEM supplemented with 15 % FBS at 37 °C in a humidified 5 % CO_2_ gassed incubator, conditions which led to doubling after approximately every 24 h. Aliquots (100 μl) of CEM and CEMvbl Cells were then counted using Nucleo Counter (Chemometec, Denmark) and the appropriate volume containing 10 million cells was transferred into eight 20 ml sterilin tubes, appropriately labelled for the following samples: CEM PZQ, VBL PZQ, CEM SQV and VBL SQV [*n* = 2].

#### Intracellular accumulation of PZQ and SQV in CEM and CEMvbl cells

The cell samples were centrifuged (2000 × *g* for 5 min at 4 °C) and the supernatant fraction discarded. A total of 10 ml of fresh DMEM media was added to the resulting pellets to give a concentration of 1 million cells/ml, and 100 μl of 1 mg/ml of both PZQ and SQV to each respective tube resulting in a concentration of 10 μg/ml. The cells were then incubated at 37 °C for 30 min in a shaking water bath. The resulting cell suspensions were then centrifuged (2000 × *g* for 5 min at 4 °C). 100 μl samples of the supernatant fraction were then removed, and the aliquots used to determine the extracellular (EXT) concentration. The excess supernatant fraction was then discarded and the resulting cell pellet washed three times in 10 ml HBSS and centrifuged (2000 × *g* for 5 min). The resulting pellets were solubilized by reconstituting in 100 μl of distilled water and used to determine intracellular (INT) concentrations as described in a method previously validated in our laboratory [29]. The experiment was carried out in quadruplicate. The samples were then assayed by HPLC, and the data expressed as cellular accumulation ratio (CAR), the ratio of the intracellular to the extracellular accumulation calculated by the formula, CAR = (INT/EXT) × 10 [intracellular concentrations were calculated using the volume of a single CEM and CEMvbl cell to be 1 picolitre (pl)] [15, 30].

#### Effect of PZQ and tariquidar on the intracellular accumulation of SQV in CEM and CEMvbl cells

In the inhibition study, the cells (CEM and CEMvbl) spiked with a concentration of 10 μg/ml of SQV were incubated alone, and in the presence of 10 μg/ml PZQ; and 1 μM tariquidar (XR) respectively.

### Data analysis

Statistical analysis was determined by Mann-Whitney U test. All results were presented as mean ± S.D. with 95 % confidence intervals for the difference between the means, where appropriate. Statistical analysis was performed by using the unpaired *t*-test and a two-tailed *P* value of <0.05 was accepted as being significant.

## Results

### Chromatography and detection

**Fig. 1:**
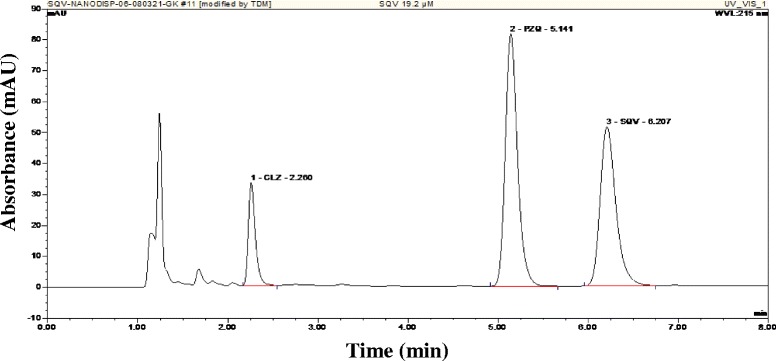
Chromatogram depicting the retention times of CLZ (internal standard), PZQ and SQV at a concentration of 19.2 μM for both drugs

The analysis of SQV and PZQ on the same mobile phase was highly dependent on the pH value of the mobile phase. Thus, the reverse-phase analysis was initially performed with various mixtures of ammonium formate, ACN and MeOH. The composition of the final mobile phase was 57 % ammonium formate, 38 % ACN and 5 % MeOH. These conditions yielded satisfactory and reproducible retention times of both PZQ and SQV. CLZ was found to be the most suitable internal standard. A sample chromatogram of SQV, PZQ and CLZ is represented in Fig. 1. The retention time for SQV was 5.1 min, whereas PZQ had 6.2 min and CLZ 2.2 min, with the total run time being 8 min. Detection at 215 nm provided adequate sensitivity.

### Linearity, limit of quantification and limit of detection

**Fig. 2:**
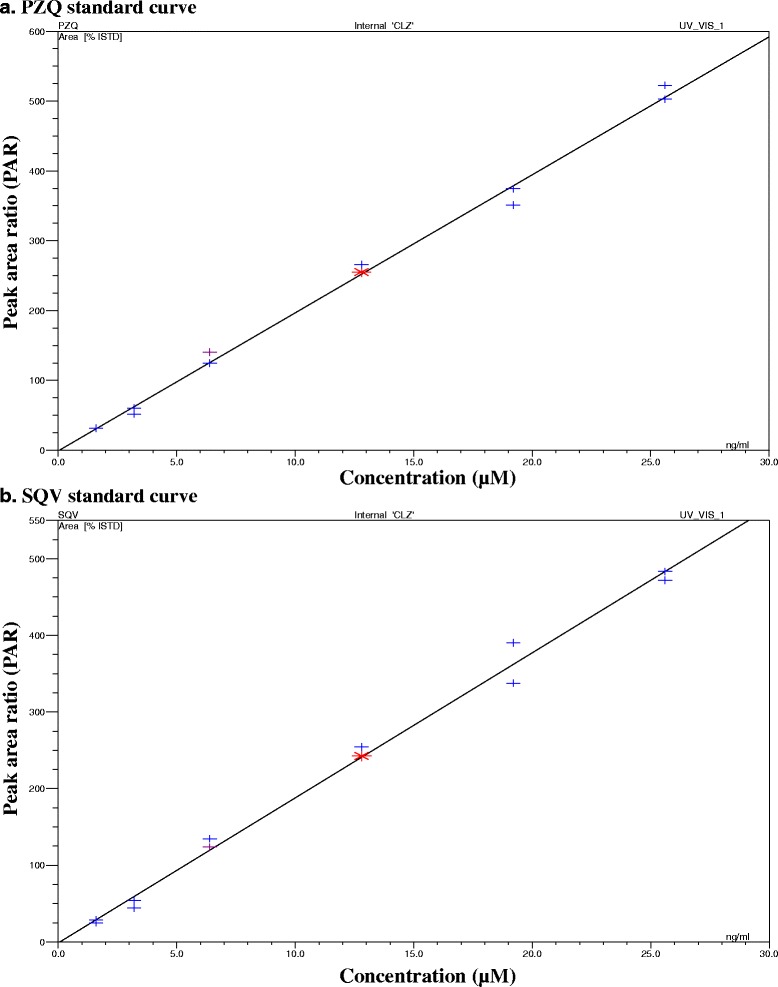
Plots of the calibration curves showing the concentration response relationships of **a** (PZQ) and **b** (SQV) on the same run

The lower limit of quantification for both SQV and PZQ on the same run was 1.6 μM whereas the upper limit of quantification was 25.6 μM. The concentration-response relationship for both SQV and PZQ standards was found to be linear in the concentration range of 1.6–25.6 μM (*r* = 0.99773 for PZQ and *r* = 0.9962 for SQV) (Fig. 2). This linear relationship was demonstrated by a coefficient of variation obtained from the daily standard curves used for the analysis of unknown samples. The lowest limit of quantification (LLQ) was 1.6 μM, while the LLD was 0.1 μM for both drugs.

### Accuracy and precision

The mean inter-day precision was within the range for both drugs with average CVs of between 3.28 and 5.89 % for PZQ, and 3.67 and 9.32 for SQV (Table 2). Similarly, the intra-day assay values were between 0.97 to 2.13 for PZQ and 0.86 to 2.15 for SQV (Table 2).

**Table 2:** Inter-day/intra-day precision, percentage recovery and stability

Drug	Inter-day precision		Intra-day precision		Percentage recovery (%)		Stability	
	Mean	CV (%)	Mean	CV (%)	Mean	CV (%)	Mean	CV (%)
PZQ								
LQC	6.29 (±0.37)	5.89	6.71 (±0.03)	3.89	106 (±14)	13.83	105 (±4)	3
MQC	12.78 (±0.58)	4.06	11.67 (±0.14)	4.06	115 (±6)	5.59	113 (±6)	5
HQC	19.38 (±0.64)	3.03	18.92 (±0.84)	4.54	115 (±3)	3.03	109 (±3)	3
SQV								
LQC	6.23 (±0.58)	9.32	6.68 (±0.58)	4.32	95 (±6)	7.28	91 (±5)	6
MQC	12.8 (±0.93)	7.24	12.61 (±0.18)	7.24	110 (±10)	9.58	100 (±5)	5
HQC	18.94 (±0.69)	3.67	19.63 (±0.91)	3.69	101 (±4)	4.46	87 (±2)	2

### Recovery

The mean recovery for both drugs in DMEM was always greater than 92 % for both drugs within the analyzed concentration range of 6.4 μM (MQC) to 19.2 μM (HQC) (Table 2).

### Specificity and selectivity

**Fig. 3:**
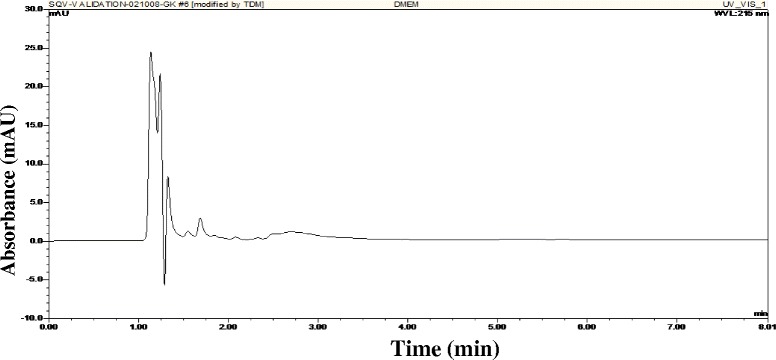
Chromatogram of the blank extract (DMEM), showing the injection peak and absence of any other interfering peaks

The selectivity of the chromatographic separation was demonstrated by the absence of interfering endogenous peaks in DMEM (Fig. 3).

### Stability

Freeze thawing DMEM samples containing SQV and PZQ did not appear to significantly affect the concentrations when compared to fresh samples (Table 2).

### Accumulation experiments

**Fig. 4:**
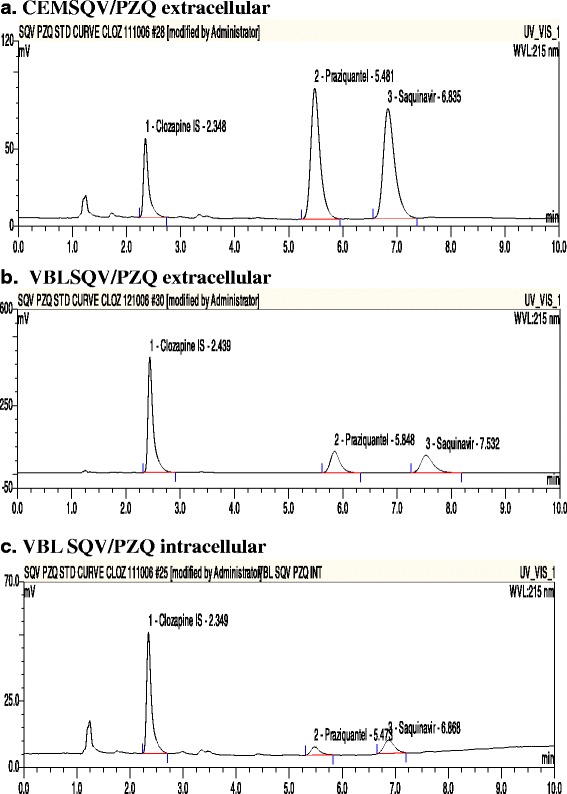
Chromatograms showing the extracellular accumulation of SQV/PZQ in CEM parental (**a**), and CEMvbl cells (**b**); and intracellular accumulation in CEMvbl cells (**c**)

**Fig. 5:**
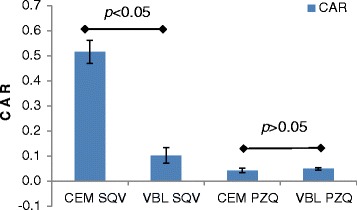
Intracellular accumulation of SQV and PZQ in CEM and CEMvbl cells, Mean ± SD (*n* = 4)

The chromatograms illustrating the extracellular and intracellular accumulation of both SQV and PZQ in CEM and CEMvbl cells are depicted in Fig. 4. The accumulation of SQV was significantly lower in CEMvbl than CEM cells (0.1 ± 0.031 versus 0.52 ± 0.046, *p* = 0.03 [*p* <0.05]), whereas similar accumulation of PZQ occurred in both cell lines (0.05 ± 0.005 versus 0.04 ± 0.009, *p* = 0.4) [Table 3; Fig. 5].

**Table 3:** Cellular accumulation ratio (CAR) values for the substrate studies

Sample	CEM SQV	VBL SQV	CEM PZQ	VBL PZQ
1	0.54	0.12	0.03	0.06
2	0.52	0.14	0.05	0.05
3	0.45	0.07	0.05	0.05
4	0.55	0.08	0.04	0.05
Mean	0.52	0.10	0.04	0.05
STDEV	0.046	0.031	0.009	0.005
*p* value	*p* = 0.03		*p* = 0.4

**Fig. 6:**
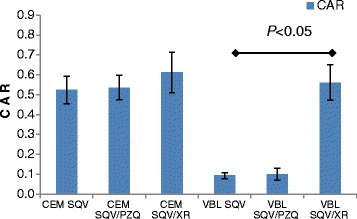
Effect of PZQ and tariquidar on the intracellular accumulation of SQV in CEM and CEMvbl cells, Mean ± SD (*n* = 4)

PZQ did not significantly affect the accumulation of SQV in either CEM (0.52 ± 0.068 versus 0.54 ± 0.061, *p* = 0.77), or in CEMvbl cells (0.09 ± 0.015 versus 0.1 ± 0.031, *p =* 0.89) cells as compared to tariquidar; 0.52 ± 0.068 versus 0.61 ± 0.102, *p* = 0.34 in CEM cells and 0.09 ± 0.015 versus 0.56 ± 0.089, *p* = 0.029 [*p* < 0.05] in CEMvbl cells (Table 4; Fig. 6).

**Table 4:** Cellular accumulation ratio values for inhibition studies

Sample	CEM SQV	CEMSQV PZQ	CEMSQV XR	VBL SQV	VBL SQV PZQ	VBL SQV XR
1	0.57	0.45	0.55	0.07	0.08	0.53
2	0.50	0.57	0.51	0.10	0.07	0.50
3	0.59	0.59	0.73	0.10	0.13	0.69
4	0.44	0.53	0.66	0.11	0.13	0.52
Mean	0.52	0.54	0.61	0.09	0.10	0.56
STDEV	0.068	0.061	0.102	0.015	0.031	0.089
*p* value		*p* = 0.77	*p* = 0.34		*p* = 0.89	*p* = 0.03

## Discussion

The purpose of this research was to characterize the P-gp substrate specificity of PZQ, a widely used drug as an attempt to understand its pharmacokinetics. This was done by investigating its intracellular accumulation in T-lymphoblastoid cells, CEM parental and CEMvbl cells which overexpress P-gp. A reversed phase HPLC method was developed for quantification.

P-gp plays an important role in the movement of many drugs across biological membranes, especially those administered through the oral route. It functions as a transmembrane efflux pump; pumping its substrates outside the cells, hence affecting its tissue concentrations and resultant pharmacological effects, including drugs interactions and resistance [31–34]. The inhibition or potentiation of the transporter function will therefore have an impact on the cellular accumulations of the drugs and efficacy, an example being the reported reduction in plasma concentrations of PIs [35–38]. In addition, the efflux protein is present in several important body tissues including the intestines, liver, kidney, brain and placenta where it has role in modulating its substrate drugs [39, 40]. P-gp along with CYP 450 group of enzymes are therefore important determinants of the pharmacokinetics of several drugs, since many are its substrates. The determination of the substrate specificity of a drug to either efflux proteins and/or metabolizing enzymes is therefore important in the understanding of its pharmacokinetics [41–44]. Indeed, the substrate specificity of several drugs to P-gp has now been characterized [32, 45].

Despite its widespread use, PZQ’s substrate specificity to P-gp has not been conclusively characterized, since authors from previous studies arrived at conflicting conclusions. Researchers from two groups concluded that it was not a substrate [19, 20], whereas another team concluded that it was an inhibitor without being a substrate [18]. However, it is noteworthy that Caco-2 cell lines used by the second group express several drug transporters (influx as well as efflux) including metabolic enzymes. An interplay of several factors is therefore possible, and careful interpretation of the results may be necessary [46–49].

In order to determine the P-gp substrate specificity of PZQ, we developed an analytical method that compared intracellular accumulations of the drug in T-lymphoblastoid cells, CEM (control) and CEMvbl which overexpress P-gp, alone and in presence of SQV, a known substrate of P-gp. Several methods have been developed to quantify PZQ levels, alone [50, 51] or in combination with other drugs [52–54]; but none so far for simultaneous determination of the drug and ARVs. We chose to use SQV, a PI as PZQ may be co-administered with PIs, since there is geographic overlap between the regions afflicted by both Schistosomiasis and HIV/AIDS [55]. The method was validated over a concentration range of 1.6 to 19.2 μM for both drugs (50 to 600 ng/ml for PZQ and 107 to 1288 ng/ml for SQV); which is within the detectable range for both drugs in human plasma [56, 57].

## Conclusion

The results from our accumulation results indicate that PZQ is neither a substrate nor an inhibitor of P-gp in T lymphoblastoid cells, CEM and CEMvbl. We also report a simple, accurate and precise method for simultaneous quantification of PZQ and SQV. Several HPLC methods have been developed for the determination of PZQ alone [50, 51], or simultaneously with other antihelminthics [58, 59]. However, to date there is none reported in the literature that has been developed for concurrent analysis of antiretroviral and antihelminthic drugs. We for the first time report a simple and accurate HPLC method for the simultaneous quantification of PZQ and SQV. This method may be used to investigate the pharmacokinetics of PZQ, including the potential drug interactions between antihelminthic and antiretroviral drugs.
